# An Anatomical Description of the Diseases of the Organs of Circulation and Respiration

**Published:** 1846-12

**Authors:** 


Art. VII.—*An Anatomical Description of the Diseases of the Organs of Circulation and Respiration.* By Charles Ewald Hasse, M.D., Professor of Pathology and Clinical Medicine at the University of Zurich, etc., etc. Translated and Edited by W. E. Swaine, M.D., Physician Extraordinary to H. R. H. the Duchess of Kent. Philadelphia: Lea & Blanchard. 1846. 8vo. pp. 377.

From the preface by the editor we make two extracts, which will afford all the information concerning this much lauded work that we have, at present, space or leisure to give. He says—

"The following pages will not be found to contain a mere descriptive catalogue of curiosities in morbid anatomy, nor records of extreme or severe cases only, but a thorough anatomical and physiological account of the origin of disease, of its progress through its several phases, and of its ultimate issue in death, in abiding organic mischief, or in recovery. The practical utility of this plan, apart from the truthfulness and ability with which it is carried out, few will be disposed to contest; for, to use the words of a highly distinguished physician of the present day,—'so far as morbid anatomy contemplates the last or latest results of disease that are fixed and irremediable, and unalterable, its value is very small. But so far as morbid anatomy contemplates *disease in progress*, and scrutinizes and explains its organic processes, its value is very great."'

Dr. Hasse was educated at Paris and Vienna.

"On his return to his own university, Leipsic, he was appointed by my revered clinical instructor, Professor Clarus, to be assistant clinical teacher, and also pathological prosector at the principal hospital. With such means at his disposal, my friend forthwith commenced forming a pathological collection, which, under his auspices, has grown into a most interesting and valuable museum; and the present work is but a collateral result of the unwearied *practical industry* which he displayed in that undertaking, combined with a thorough knowledge of all that other observers had before achieved in the same field of science.

"Hence it will at once be seen that this treatise differs essentially from what is commonly called a compilation. The high estimation in which it is held in Germany, is clearly shown by the fact that, since its publication, Professor Hasse has had the offer of the chair of clinical medicine from no fewer than five universities. He has accepted that vacated by Professor Schönlein, at Zurich, and at present holds the additional rank of Rector of that Germano-Swiss University."

